# Skills Training via Smartphone App for University Students with Excessive Alcohol Consumption: a Randomized Controlled Trial

**DOI:** 10.1007/s12529-016-9629-9

**Published:** 2017-02-21

**Authors:** Mikael Gajecki, Claes Andersson, Ingvar Rosendahl, Kristina Sinadinovic, Morgan Fredriksson, Anne H Berman

**Affiliations:** 1 Department of Clinical Neuroscience, Centre for Psychiatry Research, Karolinska Institutet, Norra Stationsgatan 69, plan 7, 113 64 Stockholm, Sweden; 2 Stockholm Centre for Dependency Disorders, Stockholm, Sweden; 3 Department of Criminology, Malmö University, Malmö, Sweden; 4 Liquid Media AB, Stockholm, Sweden

**Keywords:** Randomized controlled trial, Problem drinking, Alcohol abuse, College, University, Smartphone, Mobile phone, eHealth, mHealth, Brief intervention, Relapse prevention

## Abstract

**Purpose:**

University students in a study on estimated blood alcohol concentration (eBAC) feedback apps were offered participation in a second study, if reporting continued excessive consumption at 6-week follow-up. This study evaluated the effects on excessive alcohol consumption of offering access to an additional skills training app.

**Method:**

A total of 186 students with excessive alcohol consumption were randomized to an intervention group or a wait list group. Both groups completed online follow-ups regarding alcohol consumption after 6 and 12 weeks. Wait list participants were given access to the intervention at 6-week follow-up. Assessment-only controls (*n =* 144) with excessive alcohol consumption from the ongoing study were used for comparison.

**Results:**

The proportion of participants with excessive alcohol consumption declined in both intervention and wait list groups compared to controls at first (*p* < 0.001) and second follow-ups (*p* = 0.054). Secondary analyses showed reductions for the intervention group in quantity of drinking at first follow-up (−4.76, 95% CI [−6.67, −2.85], *Z* = −2.09, *p* = 0.037) and in frequency of drinking at both follow-ups (−0.83, 95% CI [−1.14, −0.52], *Z* = −2.04, *p* = 0.041; −0.89, 95% CI [−1.16, −0.62], *Z* = −2.12, *p* = 0.034). The odds ratio for not having excessive alcohol consumption among men in the intervention group compared to male controls was 2.68, 95% CI [1.37, 5.25] (*Z* = 2.88, *p* = 0.004); the figure for women was 1.71, 95% CI [1.11, 2.64] (*Z* = 2.41, *p* = 0.016).

**Conclusion:**

Skills training apps have potential for reducing excessive alcohol use among university students. Future research is still needed to disentangle effects of app use from emailed feedback on excessive alcohol consumption and study participation.

**Trial Registration:**

NCT02064998

**Electronic supplementary material:**

The online version of this article (doi:10.1007/s12529-016-9629-9) contains supplementary material, which is available to authorized users.

## Introduction

With the advent of the smartphone in the 1990s, manufacturers began incorporating increasingly powerful computing and communication capabilities in a handheld format, making it possible to run native applications, *apps*, and to view and interact with advanced content over the Internet. The global use of smartphones is steadily increasing and it is estimated that in 2015, there were 1.9 billion users worldwide [1]. The ubiquity and the capabilities of smartphones have spurred developers to provide content in a multitude of different areas, including health care and specifically for mental health care and substance abuse, where many apps are available but few have been evaluated scientifically [2].

Many apps related to alcohol consumption are commercially available, with the majority being for entertainment purposes, not uncommonly encouraging drinking. Of those that address reduction of drinking, only a few have been scientifically assessed [3–5]. A recent review identified only six scientifically researched apps for reducing alcohol use [6]. College and university students, who engage in more hazardous drinking and high consumption events, than do their non-student peers in the population [7, 8], are a frequent and easily accessible target group for intervention efforts. Addressing the negative consequences of hazardous drinking patterns is of particular importance to minimize both short-term negative consequences such as hangovers, aggressiveness, blackouts and worse, academic performance [9, 10], alcohol-related injury, and death [11] or the risks of developing substance use disorder in later adult development [9]. Mobile interventions could be a highly efficient medium for reaching this group. In a very recent review on mobile interventions targeting risky drinking among university students, we found only two studies that examined the use of smartphone apps [12]. In the first study, Witkiewitz et al. found that a smartphone app, containing some components of the Brief Alcohol Screening and Intervention for College Students (BASICS) program that has shown positive effects on university students’ drinking, had no effect on reducing heavy episodic drinking or simultaneous cigarette smoking and alcohol use at 1-month-follow-up [13]. The second study compared assessment-only controls with access to two smartphone apps, PartyPlanner and Promillekoll, which both offered real-time estimated blood alcohol concentration (eBAC) feedback but where PartyPlanner also offered a planning and follow-up feature. This study, conducted by our research group, found no improvement in any of the intervention groups compared to controls and a negative finding of an increase in frequency of drinking occasions (but not quantity) in the Promillekoll group. Also, we observed that about one third of the students drank excessively throughout the trial, beyond public health recommendations to drink no more than 9 standard drinks per week for women and 14 standard drinks for men. Secondary analyses revealed a gender difference: the increase in drinking frequency was found only among men [14].

**Fig. 1:**
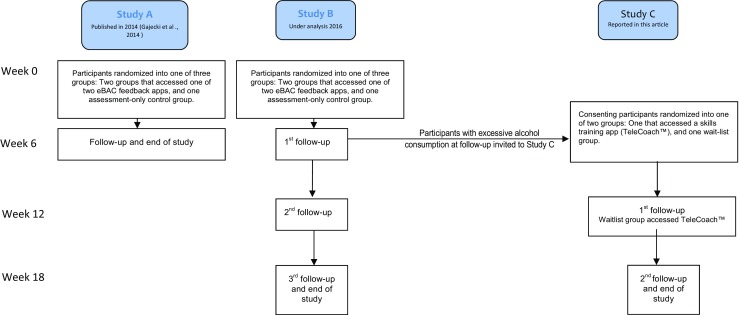
Figure comparing the time frames and flow of studies A (Gajecki et al., 2014), B (Berman et al., 2016), and C (Berman et al. 2016, and current article)

The general sparsity of research findings and the negative nature of the results in our first study (A) [14] with its 6-week follow-up led us to design two new studies (B and C) [15]. In study B, the content of both the apps tested in study A was somewhat improved and follow-up was extended to 12 and 18 weeks to detect any possible delayed effects. In study C, reported in this article, a sub-group of students with excessive alcohol consumption at the 6-week follow-up in study B was offered access to a new smartphone app with skills training components from the relapse prevention (RP) program [16], commonly used in face-to-face treatment for problematic alcohol use. For an overview of studies A, B, and C, see Fig. 1.

In the present study, our aim was to investigate whether students who did not respond to apps offering eBAC feedback might be more responsive to an app with skills training components. A fundamental part of RP consists of participants learning to identify situations where the risk of drinking is higher, and how to cope with such situations by either altering their responses to certain situations or avoiding exposure to the triggers altogether. Common coping skills taught in RP are *urge surfing*, a mindfulness exercise for dealing with urges and cravings, relaxation for coping with stress, and assertiveness skills for dealing with social pressure [17] The program in its entirety has proven effective in face-to-face treatment for alcohol problems [18]. One of the research challenges for app studies is selecting suitable components adaptable for delivery in the app format. The app used in this study packaged selected RP components in an app format and was evaluated among university students with excessive alcohol consumption.

In summary, earlier studies on smartphone apps for university students have shown null results [12] although a dose-response effect was identified where students who accessed more modules in the BASICS skills-based app were less likely to drink at all during the 14-day assessment period [13]. Given that our previous study [14] showed that about one third of the sample had excessive alcohol consumption but did not change their behavior after using a real-time eBAC-feedback-based app, we were interested in investigating the effects of giving students who reported excessive alcohol consumption, after having used a real-time eBAC app for 6 weeks (in study B), access to an in-depth skills training smartphone application, Telecoach™ as an add-on (in study C). Our primary hypothesis for study C, reported herein, was that the proportion of excessive alcohol consumption would be lower in the app group, compared to an assessment-only group. For comparison with study A [14], we analyzed alcohol-related outcomes investigated in that study. In addition, the gender differences found in that study led us to also conduct gender-based analyses in the present data. The latter were exploratory secondary analyses, not based on explicit hypotheses.

## Methods

### Design

A skills training smartphone app, TeleCoach™, was made accessible to a sample of university students with excessive alcohol consumption who already had access to one of two apps offering real-time eBAC feedback (study B) [15]. In study C, a randomized parallel three-group repeated measures design, alcohol-related outcomes for a TeleCoach™ intervention group and a wait list group were compared to an assessment-only control group. At the 6-week follow-up in study C, the wait list group was given access to TeleCoach™, and at both 6 and 12 weeks, alcohol-related outcomes were compared between the TeleCoach™ intervention group (with access to an eBAC app for 18 weeks and TeleCoach™ for 12 weeks), the wait list group (with access to an eBAC app for 18 weeks and TeleCoach™ for 6 weeks) and an assessment-only control group. The primary outcome measure was the proportion of students with excessive alcohol consumption, and secondary measures were quantity, frequency, binge drinking occasions, average eBAC per week, and peak eBAC per month, all measured at the 6- and 12-week follow-ups. The trial was registered at clinicaltrials.gov: NCT02064998.

### Participants

Two hundred fifty-seven university students from three major universities in the capital area of Sweden were invited via email to the present study if they reported excessive alcohol consumption at the 6-week follow-up in study B. The 186 participants who gave their informed consent via an online form to participating in study C were informed that they would receive a link to a new web-based smartphone application, either immediately after randomization (intervention group) or 6 weeks later (wait list group), and that they would be contacted for follow-up assessments 6 and 12 weeks later. A sub-sample from the assessment-only control group in study B, matched on excessive weekly alcohol consumption at the 6-week follow-up, was used as a control group in the present study. All participants in studies B and/or C were informed that participation with completion of all follow-ups meant that they were entered in a lottery offering the opportunity to win one of three iPads.

### Randomization

Participants in the present study were randomized to either the intervention or wait list condition, with a ratio of 1:1 using the randomization function in the IBM SPSS Statistics for MacOS X, Version 22 (IBM Corp, Armonk, NY, USA).

### Intervention

*TeleCoach™ app,* a web-based app requiring an Internet connection was developed by the authors and consists of a main menu with two parts: (a) registration of alcohol consumption in standard glasses for each day of the past week, resulting in brief feedback and information on guidelines for hazardous drinking and (b) a relapse prevention skills training menu offering two options: “say no to alcohol” or “feel better without alcohol.” The “say no” option leads to additional options for risk situation analysis or refusal exercises. The risk situation analysis consists of answering the questions from the Alcohol Abstinence Self-efficacy Scale (AASE) [19], with feedback summarizing reported risk situations. Refusal exercises are presented in text form. Selecting the “feel better without alcohol” option leads to a choice between listening to recorded relaxation exercises, positive thought exercises, or urge surfing training. Participants were instructed to use TeleCoach™ at will. They were also informed that they could continue using the app previously assigned to them in the preceding, ongoing study B.

### Adverse Event and Technical Limitations

Due to technical problems, the intervention group was given access to TeleCoach™ 3 weeks after randomization. Intervention group participants were informed about the delay about 1 week after randomization; follow-ups were scheduled for 6 and 12 weeks after access was provided, meaning that that follow-ups occurred with a 3-week time lag for the intervention group. Another technical limitation is the fact that objective data on actual app use was not available to the research group.

### Seasonality

The study took place between December 2014 and March 2015. Swedish university education is not based on the concepts of midterms or finals, so there were no uniform examination periods during this time. The weeks leading up to Christmas and New Year’s Eve are associated with parties and alcohol consumption in Sweden, and during the active study period, both major public holidays occurred. Also, follow-up data were collected for the participants in the intervention group during Easter week.

### Assessments

Baseline assessment and the two follow-ups (at 6 and 12 weeks) were conducted via an online questionnaire that included the Daily Drinking Questionnaire (DDQ) and a question on motivation to reduce alcohol consumption. Links to the assessment questionnaires were sent by email, with two reminders sent 2 days apart. The Alcohol Use Disorders Identification Test (AUDIT; [20]) was part of the baseline assessment in study B, meaning that AUDIT scores at 6 weeks prior to registration in study C were available for all study C participants. The second (and final) follow-up included the AUDIT, as well as questions on apps used, whether the participant had accessed any other means of help for alcohol consumption and questions on perceived ease of use and suitability of the app for problematic alcohol consumption, while baseline measurement and the 6-week follow-up only included the DDQ and the question on motivation to reduce alcohol consumption.

### Measures

The Daily Drinking Questionnaire (DDQ) [21] was used to measure quantity and frequency of alcohol consumption. This instrument was translated into Swedish by Malmö University in cooperation with the University of Washington. Participants were asked to consider a typical week during the last month and state how many standard glasses of alcohol they drank and over how many hours during each day of this typical week. They were also asked to report their peak alcohol consumption event during the last month in terms of how many standard glasses they drank, during a self-reported number of hours. This measure in a slightly different form has demonstrated good test-retest reliability in paper format [22] and good internal consistency (Cronbach’s *α* = .83) [13].

Estimated blood alcohol concentration (eBAC) was calculated based on the values from the DDQ in conjunction with the weight and gender of the participant. The formula used was the widely known Widmark formula as modified and used by the US National Highway Traffic Safety Administration [23]: eBAC (in parts per mille, as is standard in Sweden) = ([number of standard glasses] × 12 g) / ([body weight in kg] × C) − ([no. of hours] × 0.15), where C is a gender specific constant (0.68 for men, and 0.55 for women). In order to convert the eBAC from parts per mille to percentage values for this article, values were divided by 10.

*Motivation to reduce alcohol consumption* was measured using a simple question “How interested are you in reducing your alcohol consumption?” on a scale from 1 to 10.

### Definitions

#### Excessive Alcohol Consumption and Binge Drinking.

*Excessive alcohol consumption* was defined as drinking more than 14 standard glasses per week for men and more than 9 for women and *binge drinking* was defined as 5 or more standard glasses per occasion for men, and 4 or more standard glasses per occasion for women [24]. These definitions constitute the current recommendations of the National Public Health Agency in Sweden.

#### Standard Glass

A *standard glass* was defined as containing 12 g of pure alcohol [24].

### Outcomes

Outcomes in this study were calculated based on the participants’ DDQ registrations, with the addition of the participant’s gender and reported weight for calculating eBAC.

1. *Primary* *outcome:* the proportion of participants with excessive alcohol consumption in each group
2. *Secondary outcomes*

- *Quantity*—the number of standard glasses consumed during a 7-day period (based on the DDQ question about drinking habits in a typical week during the last month)
- *Frequency*—the number of days in a 7-day period during which the participant consumed alcohol
- *Binge occasions*—the number of days in a 7-day period where the participant engaged in binge drinking
- *Average eBAC*—the average eBAC over a 7-day period
- *Peak eBAC*—the eBAC calculated from the peak consumption event during the last 30 days

### Statistical Analyses

Descriptive statistics were used to present baseline characteristics. Analysis of variance (ANOVA) was used to determine any baseline differences between groups in age, AUDIT [20] (from baseline assessment in study B), mean eBAC, peak eBAC, quantity, frequency, and number of binge drinking occasions. Pearson’s chi-squared tests were used to determine differences between the groups in gender proportions and the proportion of participants drinking excessively. Generalized estimating equations (GEE) [25] with an exchangeable working correlation structure were used for analyses of longitudinal data: quantity, frequency, number of binge drinking occasions, mean eBAC, and peak eBAC. The semirobust Huber-White sandwich estimator was used to estimate standard errors. The sandwich estimator makes fewer assumptions than the conventional estimator of variance [26] and therefore increases the theoretical robustness of the results of GEE analyses, in relation to a possible incorrect choice of working correlation matrix [27]. All available longitudinal data were entered into the GEE analyses. No imputation was carried out as simulation studies comparing the regression coefficients and standard errors of mixed models with and without a previous multiple imputation have shown very inconsistent results [27]. In the analysis of the dichotomous outcome, no other factors or covariates were controlled for because of problems in ensuring the model would converge. We entered gender, age, and pre-randomization scores as covariates in the secondary analyses to control for possible confounding. Our assumption was that age, gender, and stability in alcohol consumption before randomization are factors correlated to the app use as well as being predictors for the outcomes. All GEE analyses were performed using Stata 14 (StataCorp. 2015. College Station, TX: StataCorp LP).

### Exclusion and Substitution

**Fig. 2:**
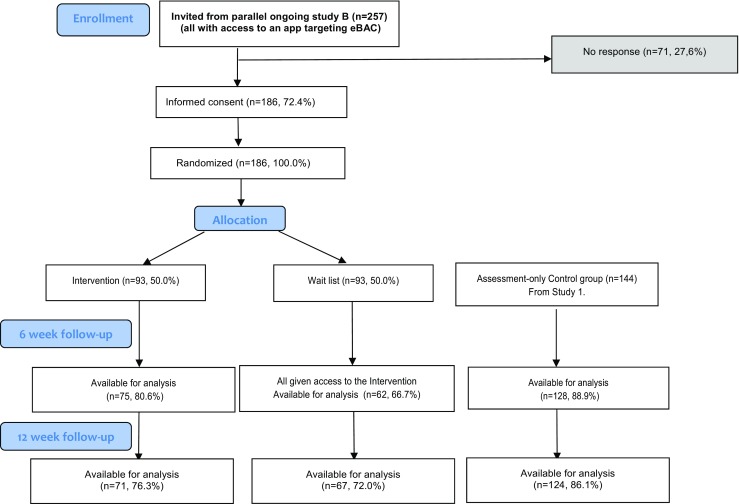
CONSORT diagram of participant flow

One participant entered clearly faulty entries in the DDQ ratings at baseline. The DDQ values for this participant were substituted with the mean sample value, as this was deemed not to interfere with the statistical calculations in any meaningful way. Other outcome variables relying on DDQ data were calculated from this substituted value (see Fig. 2 for a participant flowchart).

## Results

### Participant Characteristics

Participants were 330 university students with excessive alcohol consumption selected from a 6-week follow-up in a parallel ongoing trial. Participant characteristics at registration for the current trial, including age and gender distributions and mean scores on primary and secondary outcomes are presented in Table 1. It should be noted that AUDIT scores for the participants, available from pre-randomization, baseline assessment in study B, 6 weeks prior to the invitation to participate in the current trial, were indicative of hazardous drinking [20, 28]. Table 1 shows no overall baseline differences among participants in the three study groups. Regarding gender, over two thirds of the participants in this trial were women. Also, the average consumption in standard glasses was substantially higher at study recruitment among men (*M* = 21.92, SD = 8.40) compared to women (*M* = 14.49, SD = 5.61); (*t*(328) = −9.46, *p* < 0.001).

**Table 1:** Baseline characteristics at recruitment for students with excessive alcohol consumption in a randomized brief intervention app trial

Characteristic	Controls (*n* = 144)	Wait list (*n* = 93)	TeleCoach (*n* = 93)	Total (*n* = 330)	*p* values^b^
AUDIT^a^	14.08 (5.00)	12.69 (4.10)	13.27(4.68)	13.46 (4.69)	0.074
Women (%)	66.7	72.0	69.9	69.1	0.669
Age: mean (*SD*)	25.72 (6.75)	24.67 (6.42)	25.66 (5.99)	25.41 (6.45)	0.427
Measures of alcohol consumption: means (SD)					
Quantity (standard glasses per week)	17.16 (7.87)	16.41 (6.28)	16.58 (7.84)	16.79 (7.43)	0.716
Frequency (drinking occasions per week)	3.53 (1.39)	3.28 (1.21)	3.35 (1.20)	3.41 (1.29)	0.314
Binge occasions (number per week)	1.87 (0.86)	1.95 (0.99)	1.87 (0.91)	1.89 (0.91)	0.788
Average eBAC^c^ per week	0.037 (0.019)	0.041 (0.032)	0.035 (0.023)	0.038 (0.024)	0.227
Peak eBAC^d^ within past month	1.947 (0.960)	2.024 (1.115)	1.844 (0.904)	1.940 (0.991)	0.464
Motivation to reduce alcohol consumption (Scale 0–10)	4.85 (2.80)	4.83 (2.72)	4.83 (2.67)	4.84 (2.73)	0.997

^a^AUDIT scores were collected at recruitment to the parallel ongoing study B, 6 weeks before recruitment to study C

^b^ *P* values are based on ANOVA models for AUDIT, age, quantity, frequency, binge occasions, average eBAC, and peak eBAC while Pearson’s chi-square statistic was used for the proportion of women

^c^Estimated average percentage per week

^d^Estimated average peak percentage Blood Alcohol Count (BAC) within the past month

### Retention

Eighty-seven percent of study participants responded to at least one or both of the two follow-ups: 72.7% responded to both follow-ups, while 7.6% responded only to the first follow-up and 6.7% responded only to the second follow-up. An ANOVA analysis comparing the three retention categories and the non-responders showed no differences in baseline characteristics.

### Outcome Analyses

Regarding the primary outcome, the proportion of participants with excessive alcohol consumption was significantly higher in the control group (72.7%) compared to both the intervention group (45.3%) and the wait list group (50.0%) at first follow-up (*χ* ^2^ (2) = 17.78, *p* < 0.001) but not at second follow-up (*χ* ^2^ (2) = 5.85, *p* = 0.05). At the second follow-up, the intervention (52.1%) and wait list groups (56.7%) showed small nominal rises in the proportion of participants with excessive alcohol consumption, while a nominal decline was shown in the control group (68.5%). Across both follow-ups, the odds for not having excessive weekly alcohol consumption in the intervention group were almost twice as high as for controls, see Table 2.

**Table 2:** Excessive weekly consumption among study participants

a) Proportions (%) at two follow-up measurements				
	First follow-up*		Second follow-up**	
Group	Excessive consumption	No excessive consumption	Excessive consumption	No excessive consumption
Controls	72.66	27.34	68.55	31.45
Wait list	50.00	50.00	56.72	43.28
TeleCoach	45.33	54.67	52.11	47.89
b) Odds ratios (OR) for no excessive consumption during the whole follow-up period				
	OR^a^	95% C.I.	*Z*	*p* values
Controls	1.00			
Wait list	1.51	1.01–2.25	2.01	0.044
TeleCoach	1.95	1.36–2.80	3.63	0.000

*χ ^2^ (2) = 17.78, *p* < 0.001; **χ ^2^ (2) = 5.85, *p* < 0.054

^a^Adjusted for age and excessive consumption 6 weeks prior to registration

Participants in the current trial had already been randomized to use one of two apps with a focus on eBAC feedback in study B. As a possible confounding factor in this study could derive from eBAC app effects, we include a supplementary table showing an overview of the proportions of participants with excessive alcohol consumption in relation to their randomization in study B (Supplementary Table S1). When participants in the intervention and wait list groups were divided into sub-groups according to their study B app randomization, no differences were noted in the proportion of participants with excessive alcohol consumption. This finding was the same for intervention group participants at both follow-ups (*χ* ^2^ (1) = 0.17, *p* = 0.68; *χ* ^2^ (1) = 0.02, *p* = 0.88), as for participants in the wait list group at the both follow-ups (*χ* ^2^ (1) = 0.08, *p* = 0.78; *χ* ^2^ (1) = 0.06, *p* = 0.80).

### Secondary Outcome Analyses

We used GEE analyses to investigate time-by-group interactions, comparing the intervention and wait list groups to the reference assessment-only control group. In the analyses, we controlled for gender and age, as well as the baseline scores for each outcome parameter from study B, 6 weeks prior to the beginning of this study. As shown in Table 3, the intervention group reported reduced quantity of drinking at the 6-week follow-up and frequency of drinking at the 6- and 12-week follow-ups. These reductions were statistically significant at *p* ≤ 0.05. There were no clinically significant changes for the wait list group compared to the assessment-only control group.

**Table 3:** Mean baseline values and mean change outcome values at follow-ups, with corresponding 95% confidence intervals (C.I.) among study participants. Values are model-based and adjusted for age and specific parameter values measured 6 weeks prior to study registration

Parameters of alcohol consumption		Controls		Wait list		TeleCoach		TeleCoach vs. controls^a^	
		Mean	95% C.I.	Mean	95% C.I.	Mean	95% C.I.	*Z*	*p* values
Quantity	Baseline	16.57	15.75; 17.38	17.20	16.07; 18.34	16.71	15.38; 18.04		
	First follow-up	−2.38	−3.55; −1.21	−2.58	−4.57; −0.58	−4.76	−6.67; −2.85	*−2.09*	*0.037*
	Second follow-up	−2.49	−3.54; −1.45	−3.37	−4.98; −1.76	−3.80	−5.97; −1.63	−1.07	0.286
Frequency	Baseline	3.43	3.26; 3.62	3.36	3.18; 3.53	3.41	3.23; 3.61		
	First follow-up	−0.42	−0.67; −0.17	−0.49	−0.84; −0.14	−0.83	−1.14; −0.52	*−2.04*	*0.041*
	Second follow-up	−0.51	−0.73; −0.29	−0.63	−0.89; −0.38	−0.89	−1.16; −0.62	*−2.12*	*0.034*
Binge occasions	Baseline	1.87	1.75; 1.98	1.96	1.78; 2.14	1.86	1.69; 2.03		
	First follow-up	−0.26	−0.45; −0.07	−0.34	−0.64; −0.05	−0.52	−0.77; −0.28	−1.69	0.090
	Second follow-up	−0.25	−0.40; −0.11	−0.38	−0.61; −0.15	−0.42	−0.69; −0.14	−1.01	0.312
Average eBAC^b^	Baseline	0.04	0.03; 0.04	0.04	0.04; 0.05	0.04	0.03; 0.04		
	First follow-up	−0.00	−0.01; −0.00	−0.01	−0.01; −0.00	−0.01	−0.01; −0.00	−1.22	0.221
	Second follow-up	−0.01	−0.01; −0.00	−0.01	−0.02; −0.01	−0.01	−0.01; −0.00	−0.31	0.757
Peak eBAC^c^	Baseline	1.92	1.80; 2.05	2.09	1.88; 2.29	1.82	1.68; 1.96		
	First follow-up	−0.10	−0.26; 0.07	−0.28	−0.57; 0.01	−0.23	−0.40; −0.06	−1.07	0.283
	Second follow-up	−0.17	−0.36; 0.01	−0.36	−0.62; −0.10	−0.39	−0.54; −0.23	−1.72	0.086

^a^Only comparisons between the TeleCoach and assessment-only control groups are shown here. Wait list-control and TeleCoach-wait list comparisons did not render any significant results and are therefore not shown

^b^Per week

^c^During the last month

Secondary analyses on gender were performed on the odds ratios for not having excessive alcohol consumption, controlling for age. These analyses showed that for men, the odds ratios were 2.07, 95% CI [0.91, 4.70] (*Z* = 1.74, *p* = 0.081) in the wait list group and 2.68, 95% [1.37, 5.25] (*Z* = 2.88, *p* = 0.004) in the intervention group, both compared to assessment-only controls. For women, the odds ratios for not having excessive alcohol consumption were 1.30, 95% CI [0.82, 2.06] (*Z* = 1.10, *p* = 0.270) in the wait list group and 1.71 95% CI [1.11, 2.64] (*Z* = 2.41, *p* = 0.016) in the intervention group. This shows that the odds ratios for the intervention group were statistically significant compared to assessment-only controls both for men and for women. Additional secondary analyses on gender, controlling for age, and pre-randomization values 6 weeks before this study showed that the reductions in peak eBAC for men in the intervention group at first and second follow-ups (−0.51, 95% CI [−0.74, −0.29]; −0.63, 95% CI [−0.90, −0.36]) were significantly greater than for the assessment-only controls (*Z* = −2.32, *p* = 0.020; −0.63; *Z* = −2.19, *p* = 0.029), and also in comparison to the wait list group (*Z* = −2.80, *p* = 0.005; *Z* = −3.24, *p* = 0.001). No other significant differences were found in these secondary analyses.

## Discussion

This study investigated the effects of access to a skills training smartphone app for reducing excessive alcohol consumption. Study participants were university students reporting excessive alcohol consumption after 6 weeks of participation in a study on smartphone apps providing real-time eBAC feedback for reducing problematic drinking. The proportions of participants with excessive alcohol consumption in the intervention group as well as in the wait list group (accessing TeleCoach™ 6 weeks later) were lower than in the assessment-only group at first follow-up. Secondary outcome analyses showed significant reductions in frequency of alcohol intake at both follow-ups and in quantity at the first follow-up for the intervention group compared to both control groups. The wait list group did not differ on secondary outcomes compared to the assessment-only controls. Analyses by gender showed that men in the intervention group compared to men in the assessment-only control group had higher odds ratios for not having excessive alcohol consumption than women in the intervention group compared to women controls. Men also lowered their peak eBAC at both follow-ups compared to both the assessment-only controls and the wait list group.

This study contributes significantly to the small existing knowledge base; at this writing, very little research has been done on smartphone apps for reduction of alcohol use [6] and even less has been published on smartphone apps specifically designed for university students [12]. This study also has a somewhat longer follow-up period than the few other studies published, and it is to our knowledge the first study of smartphone apps for reducing excessive alcohol consumption in university students that has shown significant positive effects. One other study on an app featuring similar skills training to our app has been published [13], but that study targeted binge drinking college students with concurrent smoking. As far as we know, this is the first study examining a skills training app for university students, with a strict focus on problematic drinking. The present study also investigated the concept of risky drinking from several angles, primarily excessive weekly drinking, as well as binge drinking, which is known to occur more frequently among university students than non-students [7].

We found a lower proportion of excessive drinkers in both the intervention and wait list groups compared to controls, pronounced at first follow-up and marginal at the second follow-up. Indeed, the odds for not having excessive weekly drinking were twice as high in the intervention group compared to the control group and only one and a half times as high for the wait list group compared to controls. However, although we would like to ascribe these changes at first follow-up solely to the TeleCoach app, several differences between the assessment-only controls and the two intervention groups may contribute to an explanation of our results: firstly, participants in both the intervention and wait list groups were informed via email that they were drinking at hazardous levels, and that they were therefore invited to participate in an additional study. The assessment-only control group did not receive such an email. It may be that receiving this message may have influenced participants to change their drinking in the short term, and that inviting them to participate in a second study conveyed the additional gravity of the problem. A second difference is that participants in both intervention and wait list groups had had access to another smartphone app for at least 6 weeks before joining this study with continued access. As yet, we have no data on associations between the use of these apps and outcomes in this study. Our earlier study on earlier versions of the apps showed no effects after 6 weeks [14], but this does not exclude the possibility of synergistic or possible longer-term app effects.

Access to the TeleCoach™ intervention positively affected quantity and frequency of drinking patterns over 1 week, i.e., the TeleCoach™ group participants drank less alcohol up to the first follow-up and drank less often than both control groups. We found none of these effects in the wait list group, although we would have expected to see effects on quantity and frequency similar to those seen in the intervention group at the 6-week follow-up, at the 12-week follow-up, when the wait list groups had had access to the TeleCoach app for 6 weeks. It has previously been demonstrated that being in a wait list may cause participants to suspend changing their behavior until they receive the intervention [29]. However, in the present study, the wait list participants did not change on quantity and frequency after receiving access to the intervention. Instead, as previously noted, the proportion of excessive drinkers diminished in the wait list group—at rates close to those of the intervention group—at first and second follow-ups. One factor possibly influencing the differences between the groups is the fact that the control and wait list groups completed their follow-up assessments at the same time, while the intervention group completed their follow-ups 3 to 4 weeks later, due to the delayed distribution of the app. This means both that there was a greater time period between registration for the study and the first follow-up for the intervention group, and also, where seasonality effects would normally have affected all groups equally, there may have been some differences between the groups, as controls and wait list participants may have had New Year festivities included in the time frame for which they specified their drinks.

It is thus not clear whether the significant effects noted in the TeleCoach group were due to use of the app itself, receiving a message about harmful drinking in tandem with access to a skills training app, or other, unknown factors. The significant reductions on secondary outcomes in the TeleCoach group have somewhat wide 95% confidence intervals, but it could be argued that even the values at the lower end of the confidence intervals might still be clinically meaningful. The few differences in the gender-based analyses are inconclusive, but it seems that men in the intervention group had more benefit in comparison to men in the two control groups, since the intervention group men showed significant reductions on peak eBAC at both follow-ups. Men in the intervention group also had an odds ratio of 2.68 compared to men in the control group for not having excessive drinking, where women in the intervention group had an odds ratio of 1.71 compared to women in the control group. Both results are significant, but it would seem that men in the intervention group showed slightly better results. These results contrast with our previous finding, where men using the alcohol monopoly app showing eBAC feedback on drinking occasions actually increased their drinking frequency at the 6-week follow-up; these men were, however, not given any message on harmful drinking, nor any skills training components [14].

The intention behind the study design was to offer non-responders, measured by risky weekly alcohol consumption, an additional app with a focus on skills training in order to add to the possibility of addressing the excessive consumption. However, there is a possibility that actively choosing to include only individuals not responding to an app means that we mostly include individuals who do not respond to app usage, thus downplaying the potential of the app to influence their drinking.

The TeleCoach app provides a module for identifying high-risk situations as well as skills training to help participants cope with stress, urges, and social situations. Undertaking this kind of training and assessment may hinge on motivation to change one’s drinking patterns. Receiving a message about drinking excessively can raise motivation to take action. It is possible that participant motivation, not very high to begin with (average 4.84 on a scale 1–10), increased due to the excessive consumption message, and that this, together with an invitation to try another app, could largely account for our results. Future studies should include an immediate evaluation of the motivational effect of a message indicating unhealthy use, i.e., asking the simple motivation question immediately after receiving the invitation email. Our retention levels were fairly high for all follow-ups, in parity with our earlier study [14] or higher and the possibility exists that some participants replied to the follow-ups for the purpose of winning an iPad, with no actual intention of using the apps, with unclear ramifications for the outcomes.

### Limitations

There were several limitations to this study. Due to technical difficulties, we had no access to objective user data for TeleCoach™ on how and to what extent the app was used. A direct question to participants regarding their app use in the study was designed to compensate for this, but it yielded unusable data as it did not take the complexity of presenting several apps by name to research study participants into consideration. For this reason, information on actual user data—objective or self-reported—was lost. Disentangling the effects of study participation from actual app *use* is thus not possible at this stage. Questions were also asked about app usability and likeability, but these questions were also formulated as above, leading to confusing data. The fact that TeleCoach™ app required an active Internet connection could be a limiting factor, as the idea behind providing it in an app format is to make the content readily available quickly and regardless of location. For instance, access to listening to a relaxation exercise at a convenient time and place could be hindered by low connectivity.

An ethical limitation of this study is the fact that the participants in the assessment-only control group received no information on their excessive alcohol consumption. They were silently selected from the parallel ongoing study based on matching our excessive alcohol consumption definition. Nonetheless, these participants, together with all other study participants, were given a message at the end of each questionnaire to approach university student health services if they were worried about their alcohol consumption.

## Conclusions

We identified effects on excessive alcohol consumption for participants allocated to both wait list and intervention groups compared to assessment-only controls in this study. The intervention group also reduced their quantity and frequency drinking levels. The effectiveness of skills training apps for smartphones for alcohol use, both among university students and other populations, should be further researched. It is imperative to gather objective data on usage of the apps and information viewed, as well as users’ perceptions on usability of the apps to better disentangle effects of app use. An additional focus for further research on stand-alone apps would be how to design them to catch the user’s attention and maintain it, both for the duration of a behavior change cycle and for future booster sessions when the need arises. Another area of uncharted research concerns the development of apps for use in conjunction with face-to-face or digitally based care from a human treatment provider. Finally, the relapse-prevention-based skills training components offered in the in-depth app evaluated in this study should be equally effective for adult users, suggesting that a study among individuals with excessive alcohol consumption from the general population would be a valuable investment of research efforts.

## Electronic Supplementary Material

ESM 1 (DOCX 50 kb)
