# Supplementary material for: Skills Training via Smartphone App for University Students with Excessive Alcohol Consumption: a Randomized Controlled Trial
Source: Int J Behav Med. 2017 Feb 21;24(5):778–88. doi: 10.1007/s12529-016-9629-9 (PMC5608866; doi:10.1007/s12529-016-9629-9)
Supplement: Supplementary file 1 — (DOCX 50 kb) [file 12529_2016_9629_MOESM1_ESM.docx]

Table S1. Proportion of participants indicating excessive alcohol consumption by study group and prior app allocation subgroup in a parallel ongoing study (B).

| **Group/subgroup** | **Waitlist** | **Intervention** | **Control** |
| --- | --- | --- | --- |
| Total at recruitment^a^ | 100 | 100 | 100 |
| PartyPlanner at recruitment (37.6%) | 100 | 100 |  |
| Promillekoll at recruitment (62.4%) | 100 | 100 |  |
| Total at 6w^a,b^ | 50 | 45.3 | 72.7 |
| PartyPlanner at 6w | 52.6 | 48.3 |  |
| Promillekoll at 6w | 48.8 | 43.5 |  |
| Total at 12w^a,c^ | 56.7 | 52.1 | 68.5 |
| PartyPlanner at 12w | 54.5 | 53.1 |  |
| Promillekoll at 12w | 57.8 | 51.3 |  |

a. Same data as in Table 2.

b. Waitlist and intervention groups differed from controls (p<0.001; see Table 2)

c. Waitlist and intervention groups differed from controls (p=0.05; see Table 2)
